# Anatomic reconstruction of isolated left main coronary artery ostial stenosis using a right internal mammary artery patch

**DOI:** 10.1093/jscr/rjaf052

**Published:** 2025-02-11

**Authors:** Albaraa Bara, Ahmad Walid Izzat, Mohammad Bashar Izzat

**Affiliations:** Department of Surgery, Damascus University Faculty of Medicine, Mazzeh Highway, Damascus, Syria; Department of Surgery, Damascus University Faculty of Medicine, Mazzeh Highway, Damascus, Syria; Department of Surgery, Damascus University Faculty of Medicine, Mazzeh Highway, Damascus, Syria

**Keywords:** surgery, coronary, internal mammary artery

## Abstract

Surgical patch angioplasty is an alternative to classic coronary artery bypass grafting for patients with isolated coronary ostial stenosis and normal distal coronary arteries. We present a case where we successfully used an arterial patch from the right internal mammary artery to restore patency of an isolated ostial left main coronary artery stenosis. This technique is likely to offer more physiological antegrade myocardial perfusion, mimic normal vascular anatomy, and may be associated with an improved outcome.

## Introduction

Isolated stenosis of the coronary ostia associated with normal distal coronary arteries is a rare cause of myocardial ischemia, affecting < 0.2% of coronary artery disease patients [1]. In such patients, surgical patch ostioplasty has been proposed as an alternative surgical approach to classical coronary artery bypass grafting as it may provide a more physiological antegrade perfusion to myocardium and consume less graft material [2, 3].

We present a case where we successfully used an arterial patch from the right internal mammary artery to restore patency of an isolated ostial left main coronary artery stenosis.

## Case report

**Figure 1:**
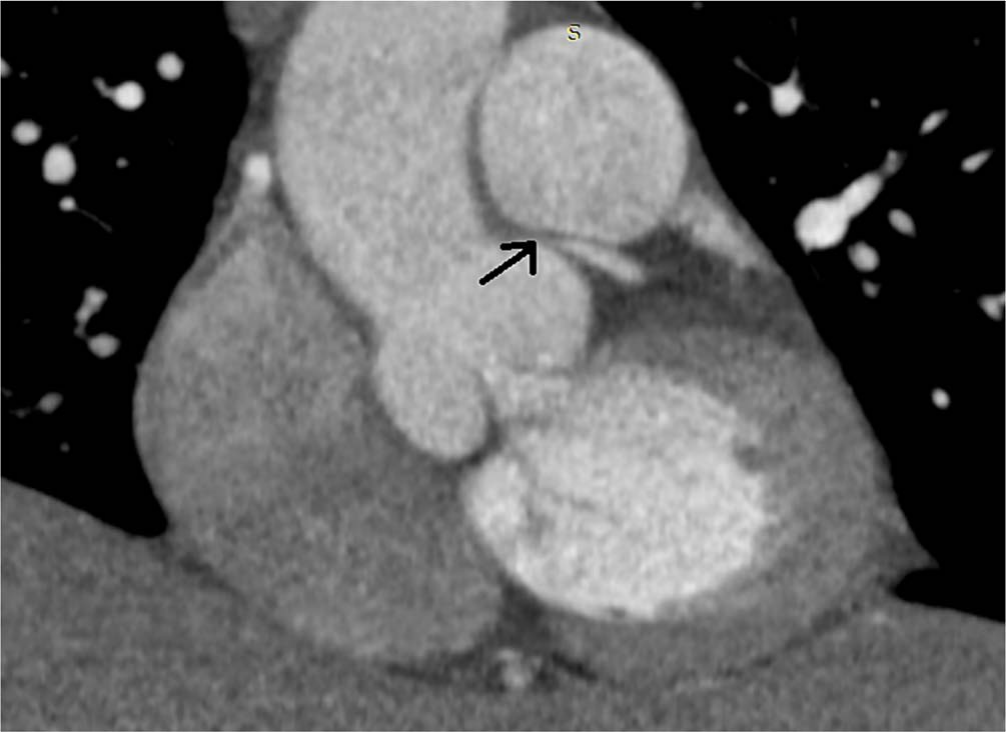
Preoperative multislice computed tomographic coronary angiography revealing sub-total occlusion of left main coronary artery, starting at the ostium and extending 5 mm distally (arrow)

**Figure 2:**
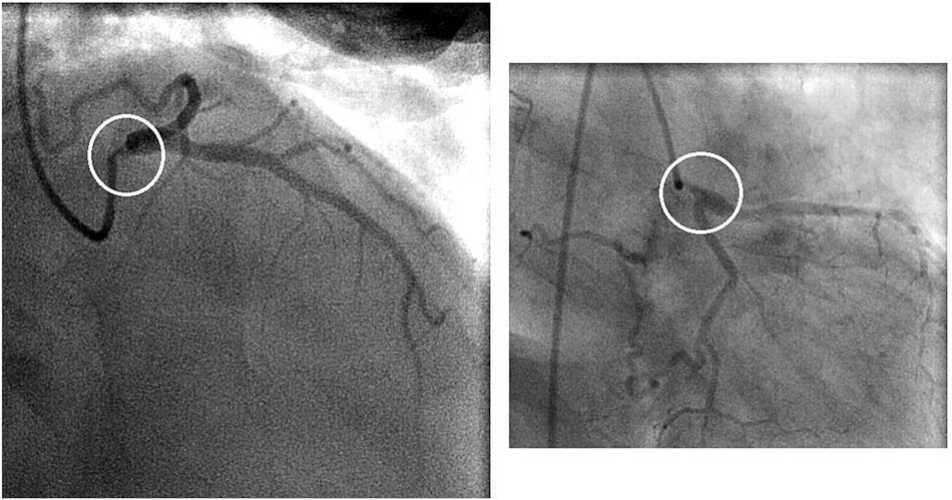
Preoperative coronary angiogram showing a 90% stenosis in the ostial and proximal shaft of the left main coronary artery (circles)

A 46-year-old female presented with recurrent chest pain radiating to the left arm accompanied by dyspnea. She was a non-smoker with no significant past medical history. Physical examination, laboratory results, chest X-ray, and electrocardiogram were all normal. Transthoracic echocardiography showed normal valvular function, a left ventricular ejection fraction of 63%, and mildly impaired diastolic function. Multislice computed tomographic coronary angiography revealed sub-total occlusion of the left main coronary artery, starting at the ostium and extending 5 mm distally (Fig. 1). Cardiac catheterization revealed a right-dominant coronary circulation, with a 90% stenosis in the ostial and proximal shaft of the left main coronary artery, with no other lesions detected (Fig. 2).

Decision to perform surgical revascularization was made. Under general anesthesia, a complete median sternotomy was performed, and the proximal segment (30 mm) of the skeletonized right internal mammary artery was harvested. Cardiopulmonary bypass was established with normothermic perfusion, and myocardial protection was achieved using del Nido cardioplegic solution. The pulmonary artery was retracted away from the aorta, and the ascending aorta was completely transected ~1 cm above aortic valve commissures. Excellent exposure of both coronary ostia was obtained, and the left ostium was confirmed to be tightly stenosed. A second incision was carried out through the aortic wall extending at a right angle through the ostium and into the roof of the left main coronary artery, reaching beyond its bifurcation well into the left anterior descending artery. Two discrete patches were produced from the internal mammary artery segment and were sutured side-to-side to create a wider patch, and were sewn with continuous 7-0 polypropylene to the aortic wall and left main coronary artery, forming a funnel to enlarge the stenosed ostia and proximal shaft. The aortic incision was closed with continuous 4-0 polypropylene. The operation and postoperative course were uneventful; the patient was discharged from the ICU after 18 hours and from the hospital after 4 days.

**Figure 3:**
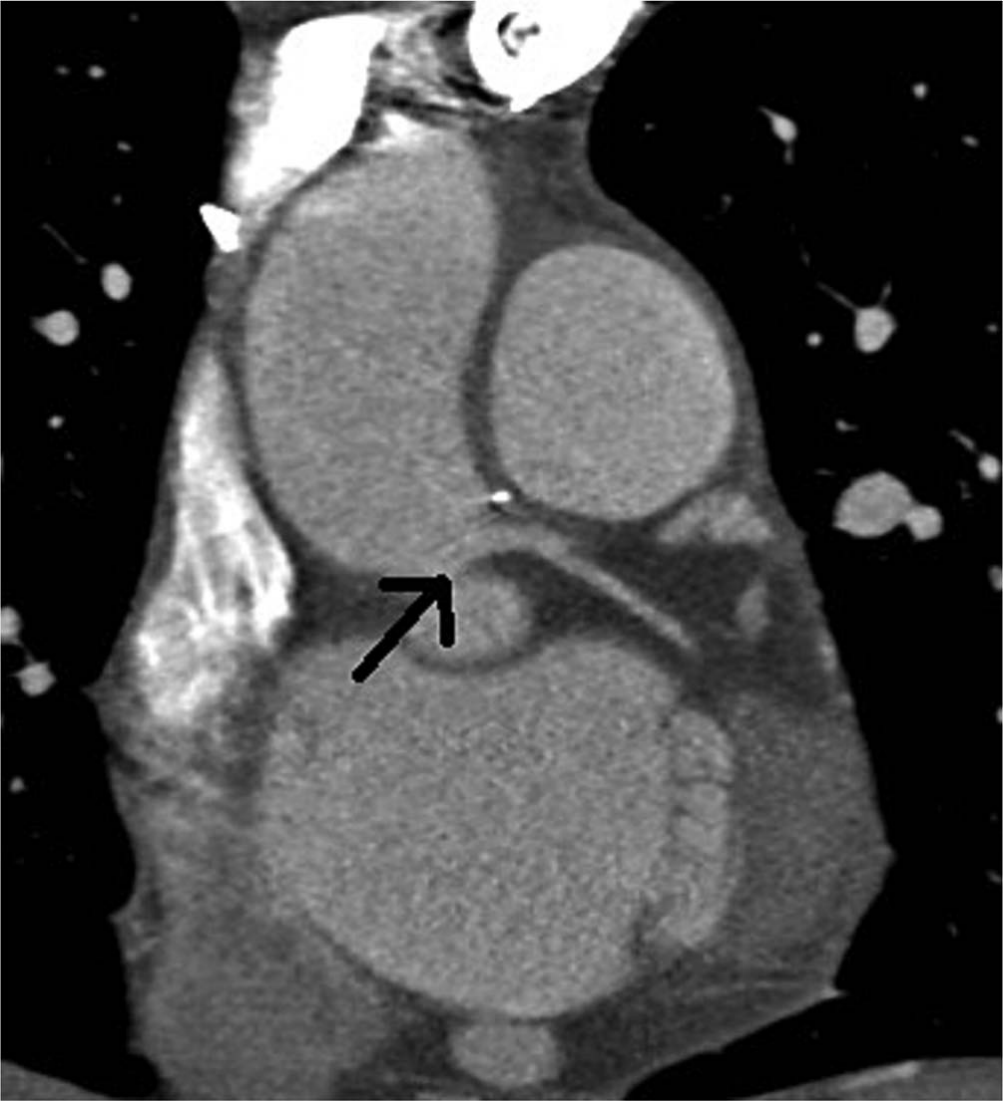
Postoperative multislice computed tomographic coronary angiography showing no signs of recurrent stenosis, with a widely open left main coronary artery (arrow)

At the 5-month follow-up, repeated multislice computed tomographic coronary angiography confirmed widely patent left ostium, with no signs of recurrent stenosis (Fig. 3).

## Discussion

Isolated stenosis of the coronary ostia is typically linked to aortic wall pathologies, most commonly atherosclerotic plaques, but may also result from iatrogenic causes, fibromuscular hyperplasia, radiation therapy, or Takayasu or syphilitic aortitis [2, 4–5].

Surgical patch angioplasty was first introduced in 1965 by Sabiston *et al.* and Effler *et al.* [6, 7], and was refined later by Hitchcock *et al.* and Villemot *et al.* [8, 9]. The most recent modification was introduced by Dion *et al.* [10] who advocated using an anterior approach whereby an aortic incision is made starting from the anterior medial wall and extending towards the left main coronary artery ostium, as we did in our case. Despite several other modifications, we consider this approach to be the most acceptable and convenient technique due to its minimal invasiveness and optimal exposure.

Compared to classical coronary artery bypass grafting, surgical patch angioplasty provides a more physiological antegrade flow pattern [11], protects the native coronary ostium from competitive flow, and allows for future percutaneous intervention if distal coronary stenosis develops [5]. On the other hand, the presence of severe ostial calcifications or extension of the disease towards bifurcation of the left main coronary artery should be regarded as relative contraindications to the application of this technique due to the potential increase in the risk of early re-stenosis.

There is no consensus regarding the optimal material for surgical patch angioplasty, hence the saphenous vein, pericardium, pulmonary artery and the ascending aorta have all been used [3, 5, 12]. Arterial patches from the internal mammary artery exhibits a close histological comparability to the native coronary arteries as well as several biological and physiological properties that act to reduce smooth muscle cell proliferation, neointimal hyperplasia and atherosclerosis, potentially reducing the risk of late re-stenosis [2].

In conclusion, this report underscores surgical patch angioplasty as an alternative for classical coronary artery bypass grafting, aiming to restore anatomical and physiological flow patterns. We also believe that the use of internal mammary artery patches is likely to be associated with improved outcomes.
